# Current Trends and Future Prospects of Biochar Use to Improve Anaerobic Digestion: An Up-to-Date Critical Review

**DOI:** 10.3390/molecules31030503

**Published:** 2026-02-01

**Authors:** Marta García-Prats, Daniel González, Antoni Sánchez

**Affiliations:** GICOM Research Group, Department of Chemical, Biological and Environmental Engineering, Escola d’Enginyeria, Universitat Autònoma de Barcelona, 08193 Bellaterra, Spain; marta.garcia.prats@uab.cat (M.G.-P.); daniel.gonzalez.ale@uab.cat (D.G.)

**Keywords:** trends, mechanisms, biochar engineering, modelling, semi-continuous, life cycle assessment, techno-economic analysis

## Abstract

Biochar supplementation has gained a lot of interest in recent years as a strategy to improve anaerobic digestion. As a result, research on the field has expanded in diverse directions, yet a clear pathway is not being followed, which can lead to unexpected or contradictory results. This review analyzed the most recent literature (2020–2024) on this topic and identified three major research trends: (i) investigating the mechanisms behind biochar enhancement of anaerobic digestion (analysis of microbial communities, interspecies electron transfer, metabolic pathways, enzymatic activity, gene expression, extracellular polymeric substances, quorum sensing, and antibiotic resistance genes); (ii) maximizing biochar applications in anaerobic digestion through the use of novel tools (biochar engineering, modeling and optimization, and integration of anaerobic digestion and other technologies); (iii) advancing towards the large-scale implementation of biochar addition to anaerobic digestion (continuous operation, biochar effects on digestate, techno-economic analysis, and life cycle assessment). By investigating these topics, key knowledge gaps and challenges to be addressed in future research were defined and discussed. This review aims to provide a clear and insightful picture of the current state and future prospects of scientific research in this field, which may be of great relevance given the current rise in this technology.

## 1. Introduction

Anaerobic digestion (AD) is a well-established strategy for waste valorization that allows the recovery of energy (biogas) and fertilizer (digestate) from a wide range of organic residues (wastewater sludge, organic fraction of municipal solid waste or OFMSW, manures and slurries, agricultural and forestry residues, etc.). European institutions have demonstrated a strong commitment to AD, as evidenced by the increase in biogas plants from 182 to 1620 between 2011 and 2024 [1] and the target to produce 35 billion cubic meters (bcm) of biomethane annually by 2030 [2]. Apart from contributing to the transition towards renewable energies, AD can help to achieve energy independence, as organic waste is a cheap and readily available substrate worldwide. Despite the many advantages, AD still faces some limitations. Firstly, biogas does not meet natural gas specifications in terms of methane purity and, therefore, must undergo costly post-treatments in order to enter the natural gas grid [3,4,5]. Furthermore, as a biological process, the efficiency and productivity of AD is susceptible to the inhibition of microbial activity by the accumulation of volatile fatty acids (VFAs), ammonia, and toxic compounds, resulting in system instability and low methane yields. These challenges hinder the establishment of AD as one of the major energy sources of modern societies comparable to other renewables like wind and solar energy.

Biochar has attracted significant attention in recent years as a potential means to improve AD. This material, originally referred to as Terra Preta [6], is the result of the carbonization of biomass in the absence of oxygen through the process of pyrolysis, and shares some of the main features of interest of other carbon-based additives (CAs) (e.g., activated carbon) [7]. Biochar production was initially developed and proposed primarily as a soil amendment for carbon sequestration [8], but soon its valuable properties led to research on its potential applications in composting, AD, soil remediation, and wastewater treatment, among others [9]. In the context of AD, the use of biochar has been associated with many advantages: increased pH-buffering capacity, the adsorption and degradation of inhibitory compounds, support for microbial biofilms, the promotion of interspecies electron transfer, the development of syntrophic relationships among anaerobic microbes, in situ biogas purification through the adsorption of CO_2_ and H_2_S and the biological conversion of CO_2_ into CH_4_, and improvement of the digestate quality as fertilizer [7,10,11,12]. These processes collectively improve the stability and efficiency of the AD system, which increases the methane yield and purity, and reduces the dependency on biogas post-treatments.

Research regarding the use of biochar to improve AD has seen a clear increase over the past few years, with the number of publications rising from roughly 300 up until 2015 to over 8500 in 2024, which has been accompanied by an expansion in the number and diversity of the research lines explored [13,14,15,16]. Parra-Orobio et al. conducted a systematic analysis to identify the main research topics on this field from 2011 to 2022 [16]. They found that before 2021, the most used keywords were “biogas”, “methane production”, “sewage sludge”, or “chicken manure” and, after 2021, new terms like “microbial community analysis”, “specific surface area”, “electron donating capacity”, and “engineered biochar” gained relevance [16]. This shift illustrates the evolution from early studies testing whether biochar could improve AD in terms of methane yield under different scenarios, to the current array of diverse research lines all seeking to go further in various directions. Nevertheless, research on the field of biochar-enhanced AD is currently not following a defined pathway, but is rather in a stage of exploration in multiple directions. Specifically, three major research trends have been identified (Table 1):

- Trend 1: investigation of the mechanisms underlying biochar-enhanced AD;
- Trend 2: use of novel tools to expand the possibilities of biochar use in AD;
- Trend 3: advancing towards full-scale biochar application in AD.

**Table 1:** Conceptual framework of major research trends and associated topics in biochar use to improve anaerobic digestion.

Identified Trends	Topics Covered by This Review
Mechanisms	Changes in microbial communities. Promotion of interspecies electron transfer. Changes in metabolic pathways. Enhancement of enzymatic activity. Regulation of functional genes expression. Secretion of extracellular polymeric substances. Promotion of quorum sensing. Suppression of antibiotic resistance genes.
Novel tools	Biochar engineering. Modeling and optimization. Integration with other technologies.
Applicability	Continuous operating conditions. Biochar effects on digestate characteristics. Technical and environmental assessment.

By investigating these topics, the main knowledge gaps at present have been recognized and used to define the challenges that need to be addressed in order for research on this field to progress and prosper. The goal of this review is to delve into the most recent and cutting-edge research regarding the use of biochar to improve AD in order to reveal current trends and future prospects. The novelty of the work resides precisely on the novelty of the articles reviewed (2020–2024), as well as the broad range of fields of expertise covered (microbiology, omic sciences, bioinformatics, industrial engineering, agronomy, technoeconomic and environmental assessment, etc.). Moreover, although there are high-quality papers addressing some of the topics discussed in this review, such as the microbial mechanisms affected by biochar addition [17], the use of modeling and life cycle tools [13], or the integration of AD and biochar production [18,19], to the knowledge of the authors, there is no review in the literature that includes all three trends: underlying mechanisms, the use of novel tools, and efforts towards large-scale application. This review aims to provide a clear and insightful picture of the current state and future prospects of scientific research on biochar-enhanced anaerobic digestion, which may be of great relevance given the current rise in this technology.

## 2. Trend 1: Mechanisms Behind Biochar Improvement of Anaerobic Digestion

Many works have discussed the ways in which biochar can enhance AD performance [7,10,12], but fewer delve into the microbiological dimension behind those improvements [17]. The studies compiled on this trend have studied the changes on a genetic, enzymatic, metabolic, and microbial level that occur in AD systems supplemented with biochar and could be the reason for their improved performance. The most commonly analyzed parameters are the composition of microbial communities and the promotion of electron transfer mechanisms. This section focuses mainly on recently proposed mechanisms, including the regulation of relevant metabolic pathways, functional genes, and enzymes; the secretion of extracellular polymeric substances (EPS) and signal molecules related to quorum sensing (QS); and the suppression of antibiotic resistance genes (ARGs). Alterations at genetic, enzymatic, metabolic, and microbial levels are strongly interconnected to one another and differ based on the specific conditions of each AD system. Relevant parameters conditioning these mechanisms include the origin and pretreatment of the waste, the biomass, pyrolysis conditions, and potential modification methods used for biochar production, and the conditions of the AD process like the temperature range or the presence of inhibitors, to name a few. This should be considered when addressing the main causes of biochar effects on AD, although it is not the focus of this section.

### 2.1. Changes in Microbial Communities

Biochar possesses a highly porous structure and, therefore, has high adsorption and immobilization capacities. The feedstock used and the pyrolysis process determine the number, size, and distribution of the pores, as well as the total specific surface area (SSA). Considering the average size of biochar pores and methanogenic archaea, it has been estimated that each pore of biochar could accommodate from two to tens of methanogenic cells [20]. Consequently, a larger SSA and porosity are generally correlated with higher methane yields in AD. These properties can be maximized through activation methods to enhance biochar benefits in AD (Section 4.1). Furthermore, biochar has been found to selectively increase the abundance of microorganism species related to interspecies electron transfer mechanisms [10]. The most commonly enriched bacterial species in response to biochar addition are *Bacteroidetes*, *Geobacter*, *Firmicutes*, *Proteobacteria*, and *Actinobacteria*, while the main archaea are *Methanosarcina*, *Methanosaeta*, and *Methanobacterium* [21]. Microbial enrichment can improve AD performance in many ways other than increasing methane yield and solids removal, which include shortening the lag phase, accelerating recovery rate, or improving the resistance to inhibition.

### 2.2. Promotion of Interspecies Electron Transfer

The efficiency of the AD process is heavily influenced by the syntrophic interactions between acetogenic bacteria and methanogenic archaea, which depend on electron transfer mechanisms. Indirect interspecies electron transfer (IIET) takes place through soluble (acetate, formate, hydrogen) and insoluble (humic compounds) substances, and direct interspecies electron transfer (DIET) can occur via membrane-bound transporter proteins, electric conductive pili, and conductive materials (e.g., biochar) [10]. Biochar properties, including high EC, aromatic structure, and redox-active surface functional groups, promote the exchange of electrons between microbial cells through DIET [21]. Moreover, a large SSA and porosity favor the immobilization of microorganisms on biochar’s surface, which reduces the distance between microbes and facilitates IIET [7]. Again, biochar activation methods can be used to enhance their role in interspecies electron transfer (Section 4.1). All these improvements result in enhanced organic matter removal and methane production.

### 2.3. Changes in Metabolic Pathways

The analysis of metabolic pathways can help us to understand the improvement of AD performance mediated by biochar. The Kyoto Encyclopedia of Genes and Genomes (KEGG) is the database most commonly used for the biological interpretation of metagenomic sequence data. KEGG comprises six Level 1 categories (metabolism, genetic information processing, environmental information processing, cellular processes, organismal systems, and human diseases) with their respective subcategories in Levels 2 and 3. Biochar addition primarily promotes metabolism, increasing the relative abundance of metabolism-related functions in Level 2 like carbohydrate metabolism, amino acid metabolism, and energy metabolism [22,23,24,25]. More specifically, biochar enhances Level 3 central metabolic pathways including glycolysis/gluconeogenesis, the citric or tricarboxylic acid (TCA) cycle, and pyruvate, propanoate, butanoate, and methane metabolism [22,23,25,26,27].

Metagenomic analysis of the main AD phases can be performed in order to identify which pathways are mostly promoted by biochar. Acetogenesis pathways include homoacetogenesis (HOM), syntrophic propionate oxidation (SPO), and syntrophic butyrate oxidation (SBO). Biochar addition was found to especially augment SPO and SBO, which was consistent with an enrichment in syntrophic bacteria and the enhanced consumption of propionate and butyrate to alleviate VFA accumulation [28]. Regarding methanogenesis, different scenarios have resulted in a varied promotion of acetoclastic, hydrogenotrophic, methanol-based methylotrophic, and methylamine-based methylotrophic pathways [22,25,27,29,30]. These preferences in metabolic pathways are the result of changes in the activity of key enzymes and the expression of functional genes, which are often confirmed with changes in microbial communities.

Biochar also upregulates functional proteins in other Level 1 categories related to the biosynthesis of amino acids, membrane transporters, translation, replication and repair, folding, sorting and degradation, quorum sensing, and ribosomes [24,31,32]. In conclusion, biochar supplementation can effectively promote microbial growth and activity, and accelerate relevant metabolic pathways in AD, thus increasing the degradation of organic substrates and promoting biogas production.

### 2.4. Enhancement of Enzymatic Activity

**Figure 1:**
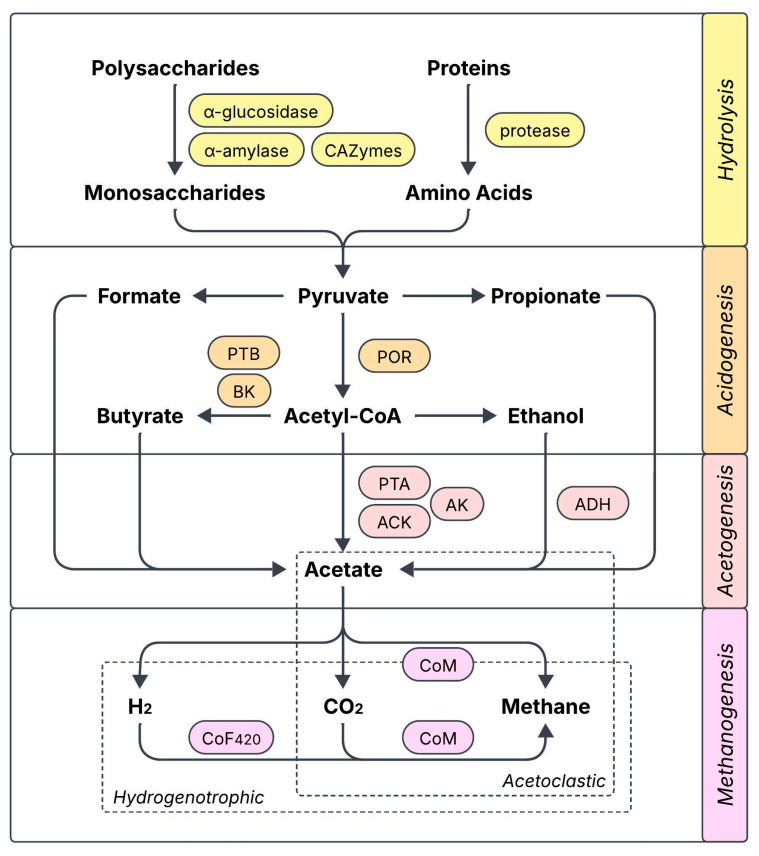
Key enzymes and co-enzymes related to anaerobic digestion that have been reported to be enhanced by biochar supplementation (CAZymes: carbohydrate enzymes, POR: pyruvate ferredoxin oxidoreductase, PTB: phosphate butyryltransferase, BK: butyrate kinase, PTA: phosphate acetyltransferase, ACK: acetate kinase, AK: adenylate kinase, ADH: ethanol dehydrogenase, CoF420: coenzyme F420, CoM: coenzyme M).

Several works relate biochar supplementation with a boost in the activity of key AD-related enzymes, which would explain the accelerated degradation of organic components and the subsequent increase in methane production. The reported cases have been compiled in Figure 1 and include enzymes participating in hydrolysis (α-glucosidase, α-amylase, protease, CAZymes: carbohydrate-active enzymes), acidogenesis (POR: pyruvate ferredoxin oxidoreductase, PTB: phosphate butyryltransferase, BK: butyrate kinase), acetogenesis (PTA: phosphate acetyltransferase, ACK: acetate kinase, AK: adenylate kinase, ADH: ethanol dehydrogenase), and coenzymes from several methanogenesis enzymes (CoF420: coenzyme F420, CoM: coenzyme M) [27,32,33,34,35,36,37,38,39]. In many cases, the activity increase can be correlated with an enhanced expression of the genes encoding for these functional enzymes. The role of biochar in enhancing enzymatic activity has been largely attributed to a greater bioavailability of trace elements (e.g., Fe, Co, Cu, Ni, Zn) that are essential for the synthesis of the enzymes, which could either be released from biochar or made more available through it [33,34,38]. Modified biochar can further increase enzymatic activity compared with regular biochar [32], although it has also been found to decrease the activity of some key enzymes at excessive doses [33]. The modulation of enzymatic activity also depends on the environment, as Yu et al. found that the activity of CAZymes was incentivized by biochar during the initial steps of AD, and later decreased once substrate availability diminished and methanogenesis took place [27].

Biochar’s promotion of enzymatic activity has many benefits on AD performance, apart from the obvious increase in methane yield. Shao et al. identified the enhancement of AD-related enzymes/coenzymes as one of the reasons why a biochar-doped semi-continuous system could resist higher organic loading rates (OLR up to 7 g VS L^−1^ d^−1^) than the control (6 g VS L^−1^ d^−1^) [37]. Apart from AD-related enzymes, biochar can regulate antioxidant enzymes like glutathione reductase and superoxide dismutase to ameliorate intracellular oxidative stress [37]. Electron transport system (ETS) activity has also been found to increase with biochar addition [33,34,35], as well as cytochrome c activity [32], implying that biochar has a greater potential to mediate electron transfer. In inhibited AD systems, biochar can boost enzymes involved in the degradation of the responsible contaminants to relieve stress [40]. Furthermore, the promotion of basic enzymes for energy supply like ATPases could facilitate other energy-demanding processes like the synthesis and secretion of EPS and the degradation of pollutants [28].

### 2.5. Regulation of Functional Genes Expression

One way of detecting changes in microbial behavior is analyzing the expression of functional genes, which are mostly related to substrate utilization, metabolic pathways, electron transfer, and the organization of microbial communities. The upregulation of functional genes associated with carbohydrate, amino acid, and lipid metabolism accelerates the degradation of organic substrates, which explains the improved hydrolysis and acidogenesis efficiencies observed in biochar-amended AD systems. Yu et al. found that the biochar-induced upregulation of several genes related to the production of acetic, propionic, and butyric acid, thus enhancing VFA production during acidogenesis [27]. Shi et al., on the other hand, reported an increase in the expression of genes linked to propionate and butyrate consumption, which alleviated VFA accumulation [28]. Biochar has also been found to regulate genes related to methanogenesis pathways. Under ammonia inhibition, Wang et al. observed a clear upregulation of coenzyme F_420_ hydrogenase, which allows electron transfer in CO_2_ to CH_4_ conversion. This indicates that hydrogenotrophic methanogenesis is suppressed under ammonia inhibition, and biochar alleviates this pressure by ensuring enough abundance of the responsible genes [41]. Zhang et al. also recognized an upregulation of enzymes related to CO_2_ reduction to CH_4_ that confirmed a switch from acetoclastic to hydrogenotrophic methanogenesis as ammonia stress increased [42]. The role of biochar in ameliorating inhibitory conditions through the regulation of gene expression is supported by works on AD systems limited by ciprofloxacin [43] and phenanthrene [44], where the addition of biochar enhanced the expression of enzymes responsible for the degradation of these compounds. Last, biochar has been seen to increase the expression of genes encoding for conductive pili (*pilA*) and flavoproteins (*fixA*), pointing at the promotion of DIET as another reason for the increase in methane yield [45].

Results on functional gene expression can be related to changes in microbial populations. For instance, Zhang et al. correlated the downregulation of enzymes involved in acetoclastic methanogenesis with a decrease in the abundance of acetoclastic archaea Methanosaeta [42]. Last, it is believed that the improvement in energy obtention allows microorganisms to dedicate resources to other processes apart from cell growth such as microbial organization. Biochar has been found to induce the expression of signaling molecules related to QS that are responsible for EPS secretion and biofilm formation [35,46]. Ma et al. reported an increase in genes responsible for membrane transport, which could be secreting compounds forming EPS, thus enhancing AD through biofilm formation and improved electron transport [47].

### 2.6. Secretion of Extracellular Polymeric Substances

EPS are polymers of high molecular weight secreted by microorganisms that intervene in the formation of microbial biofilms. They maintain the structure of the biofilm, act as a physical barrier to protect microbes from inhibitory compounds, and contain redox-active compounds that mediate interspecies electron transfer [17]. Moreover, aggregated biomass can be retained in the digester for longer times than suspended microbes, avoiding washout. Biochar is believed to release toxic compounds (e.g., metal ions, free radicals) to the aqueous phase of the digester that induce EPS secretion by microbial communities to protect themselves [48]. This has been reported to have a positive effect on methane yield on several occasions [49,50]. One reason for this would be the correlation between EPS secretion and the enrichment of microbial DIET-partners like Clostridium and *Methanobacterium* [50]. The role of EPS is especially important when AD is combined with electrochemical systems [35] (Section 4.3.2). However, passed a certain threshold or when combined with nanomaterials, biochar can collapse the EPS defense system, thus weakening redox-mediated reactions, reducing microbial activity, and overall hindering AD performance [51].

Biochar not only increases EPS secretion but can also modify its properties. Hu et al. reported that the addition of magnetic biochar increased tightly bound EPS (TB-EPS), which plays a more important role in sludge aggregation than loosely bound EPS (LB-EPS) [52]. This was confirmed by Nabi et al., who in addition investigated changes in the protein and polysaccharide composition of the EPS fractions [53]. Biochar can improve the electrochemical properties of EPS in many ways, such as increasing its capacitance and decreasing electron transfer resistance [54], increasing the presence of redox-active compounds [55,56], or promoting the formation of conductive pili [48]. Last, biochar reportedly reduced EPS secretion under toxic pressure by antibiotics ciprofloxacin [43] and tetracycline [57]. This could indicate that biochar avoids the excessive secretion of EPS, or enables EPS consumption as the carbon source, which would also explain the increase in methane production.

### 2.7. Promotion of Quorum Sensing

QS is a system of intercellular communication mediated by signal molecules like acylated homoserine lactones (AHLs), antipeptide (AIP), autoinducer-2 (AI-2), cyclic diguanosine monophosphate (c-di-GMP), diffusible signal factor (DSF), and signaling peptide (NprX) [46]. These molecules control microbial behavior during biofilm formation by regulating gene expression, metabolic pathways, and EPS secretion [17]. Biochar supplementation to AD systems has been proven to increase the concentration of QS signal molecules [58], as well as the expression of related genes and transcription factors [35,46]. Chen et al. found that higher AHL levels were achieved with the co-addition of exogenous AHL and biochar than AHL alone [59]. In a similar study, Li et al. obtained a bigger increase in methane production with AHL and biochar co-addition than with AHL and biochar separately (41.7, 35.2 and 10.3% more than control, respectively) [60]. In summary, the increase in interspecies microbial communication through QS fits in with the other beneficial effects on AD attributed to biochar, such as facilitating biofilm formation, enriching electro-active microorganisms, promoting interspecies electron transfer, and accelerating the metabolic reactions involved in organic matter degradation.

### 2.8. Suppression of Antibiotic Resistance Genes

ARGs are environmental pollutants that pose a serious health risk because they can be horizontally transmitted between microorganisms through mobile genetic elements (MGEs) (e.g., plasmids, integrons, and transposons) [17]. Some organic wastes, especially sewage sludge and animal manures, contain large amounts of ARGs (e.g., *sul*, *tet*, *bla*, *erm*, *fca*, *mdr*), which can be partially removed using treatments like AD. Biochar has been proposed as an additional strategy to contain ARGs and minimize the risk of resistance dissemination into the environment. A biochar-mediated reduction in ARGs has been largely linked to the decrease in the abundance of potential ARGs host bacteria, including *Chloroflexi*, *Bacteroidetes*, *Firmicutes*, *Acidobacteria*, *Proteobacteria*, and *Lactobacillus*, among many others [61,62,63]. ARGs can also be removed through adsorption made possible by the large SSA and highly porous structure of biochar [64,65]. Apart from reducing the overall presence of ARGs, biochar can minimize their transference by removing MGEs (e.g., *intl1*) [62,63,64], although a study performed on sludge AD found a relevant increase in MGE abundance when adding magnetic biochar [66]. Finally, the positive effects of biochar in alleviating stressful conditions decrease microbial selective pressure, which slows down ARG transformation [37,67]. In relation to this, biochar supplementation is known to improve the soil conditions (e.g., carbon sequestration, water holding capacity, nutrient content, porosity) and, therefore, could also reduce the selective pressure and spread of ARGs in the soil–plant system [68].

## 3. Trend 2: Use of Novel Tools to Expand Biochar Applications in Anaerobic Digestion

This section covers various research lines that apply complementary technologies or new approaches to maximize the beneficial effects that have been attributed to biochar addition to AD. The physicochemical properties of biochar can be improved by means of activation methods or the combination with nanomaterials, thus broadening the functionalities of this material and allowing specific targets to be met. Mathematical models have recently been applied to biochar-amended AD to better understand the process and optimize key operational parameters. Once trained and validated, the more complex models are capable of making predictions upon unforeseen data, which can help research in the field to advance faster and more efficiently. Finally, the integration of AD with other technologies can contribute to improving circularity in organic waste management and valorization, and could be the most viable way to apply biochar supplementation to full-scale AD.

### 3.1. Biochar Engineering

The most relevant properties of biochar for AD applications include SSA, porosity, cation exchange capacity (CEC), electrical conductivity (EC), and surface functional groups. These characteristics are determined by the feedstock and the pyrolysis process, which can be engineered to obtain a material with the desired features [69]. However, pristine biochar possesses restricted properties that limit its potential in AD. Activation and modification methods allow the generation of a wide range of functional biochar types that can be tailor-made for each desired purpose. The most common methods used to functionalize biochar for AD applications are summarized in Table 2. These can be classified as physical (steam, CO_2_, ozone, air, O_2_), chemical (H_2_O_2_, acid, alkaline and metal agents, heteroatom doping), and biological (microorganisms and enzymes) [7,70]. They can either be applied to the feedstock biomass, the pyrolysis process, or the biochar produced.

#### 3.1.1. Physical Methods

Physical methods involve physical oxidizing agents including steam [71], CO_2_ [72], ozone [73], air, and O_2_. The gaseous streams, typically at high temperatures, penetrate the structure of biochar and gasify carbon atoms, thus opening and widening pores and increasing its SSA and porosity. It also increases the abundance of oxygen-containing functional groups. The increase in SSA and porosity favors microbial biofilm formation, while the presence of redox functional groups improves EC and promotes electron transfer mechanisms. Microwave-assisted pyrolysis (MWAP) integrates microwave radiation in the pyrolysis process to maximize biomass disintegration [103]. Zeynali et al. achieved a 50% increase in cumulative methane production when using a MWAP-activated biochar compared with pristine biochar [74]. Physical methods are simple and inexpensive but are energy consuming.

#### 3.1.2. Chemical Methods

Chemical methods use chemical oxidizing agents like acid, alkaline, or metal solutions. Similarly to physical methods, they increase SSA and porosity through creating and enlarging pores, and generate oxygen-containing functional groups, but can also introduce more specific modifications. After activation, the biochar has to be thoroughly washed to neutralize its pH and remove any remaining chemicals, which consumes a lot of water and hinders the environmental sustainability of these methods [7].

One of the most commonly used oxidizing reagents is H_2_O_2_ [75,76]. Acid activation is carried out using H_3_PO_4_ [76], H_2_SO_4_ [77,78], HNO_3_ [79,81], and KH_2_PO_4_ [64,80] solutions. They increase the content of acidic functional groups on the surface of biochar, which has been reported to favor the CO_2_ and ammonium adsorption capacity of biochar, thus aiding AD with biogas in situ purification and ammonia inhibition mitigation. Alkali treatments involve KOH [82,83], NaOH [85,104], and K_2_CO_3_ [84] and can promote the aromatization of biochar.

The introduction of metallic compounds improves the redox properties of biochar, which accelerates microbial activity and degradation, and can alleviate ammonia and VFA inhibition [62,105]. Iron can be impregnated onto the surface of biochar in ionic form using metallic solutions like FeCl_3_ [45,90], in oxidized form using iron oxides [88,89,92,93], or ains reduced form using nano zero-valent iron (nZVI) [25,76,86,87], among others. The combination of biochar and nanomaterials has recently gained a lot of interest due to nZVI’s strong reducibility and capacity to convert CO_2_ into CH_4_, and the convenience of recycling magnetic biochar with a magnet [106]. Other metals often paired with biochar are cobalt [29] and manganese [91]. The addition of multiple metals has been seen to be advantageous over single-metal modifications due to their synergistic effects [95,96,98].

Another chemical modification is heteroatom doping, which consists of introducing groups containing elements like N, P, and S. Nitrogen-doping is the most widely applied and is known to improve EC and electron transfer at the interface of biochar. Just as in metal impregnation, dual-heteroatom doping (e.g., N, S co-doping) can achieve synergistic effects compared with single-heteroatom doping [77,94]. Chemical methods can be combined to maximize biochar modifications. Ning et al. performed an acid pre-treatment with H_3_PO_4_ to increase biochar SSA and porosity before loading nZVI [76], while Li et al. created oxygenated groups with EPS-doping prior to nZVI [91]. Coupling of metal impregnation and heteroatom doping has also been reported [97,99].

#### 3.1.3. Biological Methods

The exogenous incorporation of bio-additives like enzymes and microorganisms can improve AD performance by accelerating organic waste degradation, protecting microbes from inhibitory conditions, and improving the overall system stability and efficiency [11]. Immobilization allows their retention inside the digester, but the competitive environment of the AD system limits the time period in which they remain active, which is the main bottleneck of these methods. Biochar-supported bioaugmentation can be performed with specific microorganism species like *Methanosarcina thermophila* [100,101] or mixed syntrophic consortiums [102], which can be acclimated from the AD system itself. To the knowledge of the authors, no studies reporting the application of biochar-supported enzymes on AD are available in the literature.

### 3.2. Modeling and Optimization

Mathematical models are applied to AD with three main goals: (i) understand the behavior of the AD system by identifying key parameters and their interactions, (ii) predict the outcome of the AD process under various conditions, (iii) optimize operational parameters to enhance AD performance [107]. Moreover, the use of modeling and optimization tools reduces experimental workload, saving time, costs, and resources. In general, mathematical models (Table 3) are classified as mechanistically inspired models, kinetic models, and phenomenological models. When going from mechanistically inspired to phenomenological models, the amount of experimental data needed increases, while the use of theory decreases [107].

#### 3.2.1. Mechanistically Inspired Models

Mechanistically inspired or white-box models rely on the physical, chemical, and biological processes of AD to simulate its behavior. Anaerobic Digestion Model No. 1 (ADM1) was developed by the International Water Association (IWA) and is the reference AD structured model. It is a very complete and complex model with highly accurate predictions but the limited knowledge on microbial communities and metabolic pathways still challenges its calibration/validation under different AD conditions [125]. To date, ADM1 has not yet been applied on biochar-amended AD, as biochar’s effects on AD process are still largely unknown.

#### 3.2.2. Kinetic Models

Kinetic or gray-box models use simplified equations that relate AD performance to input data. They assume that microbial concentration is the only factor responsible for biogas production, and that microbial growth is only limited by hydrolysis rate and substrate availability. They can be single or multiple stage depending on the number of models that are applied during the digestion period, and are generally based on the first-order model (FOM), logistic model (LM), transference model (TM), or modified Gompertz model (MGM). These models allow the determination of parameters such as lag phase (d), maximum biogas/methane production (mL or mL g^−1^ VS), and maximum biogas/methane production rate (mL d^−1^ or mL g^−1^ VS d^−1^). MGM is the most extensively used and has been proven to accurately (R^2^ > 0.9) model AD performance with biochar addition in both mono-digestion [61,110,113] and co-digestion [109,112] scenarios. Some studies have compared several kinetic models obtaining various results [73,108,111].

#### 3.2.3. Phenomenological Models (Machine Learning)

Phenomenological or black-box models are built around basis functions with unknown parameters with no physical meaning, which are determined using large amounts of experimental data. They are based on machine learning technologies that require training, validation, and testing stages. Their main advantage is that they can make predictions without knowing the complex interactions per se, so they are convenient when the relationship between input and output parameters is unknown, the key parameters are not clear, and the equations cannot be explained or need assumptions [126]. The machine learning algorithms mostly applied to model the AD process with biochar are artificial neural network (ANN) and random forest (RF). These can be coupled with design of experiments (DoE) techniques like Box–Behnken or central composite designs to reach the most appropriate answer with the least number of experiments [119].

ANNs were created using the human brain as inspiration. Briefly, an ANN connects a number of independent and response variables through a set of functions, which are optimized by comparing calculated and experimental values until a minimum error is reached. ANNs have been used in AD to study the impact and interactions of many parameters covering feedstock characteristics (TS, VS, and substrate-to-inoculum ratio), biochar properties (surface area, pore volume, pore diameter, and C, H, N, and O content), and operational conditions (biochar dosage, temperature, and time) [117]. They have also been used to optimize operational parameters for maximum methane yield, with biochar dose, C/N ratio, and solid loading being the most common [114,115,119]. The latter reported a clear outperformance of ANN over the conventional GMG. RF models combine the outcomes of a series of decision trees to generate the final prediction. They have been used to assess the impact of biochar properties and operation conditions on methane production [120] and to forecast whether DIET dominantly influences methane generation with biochar addition based on its properties [118]. Last, automated machine learning algorithms are capable of functioning automatically, saving time, and being easier for non-experts to handle. They have also been applied to study the effect of biochar’s properties and operating conditions on methane yield, revealing that the solids content in the digester is crucial for selecting the biochar with appropriate characteristics [116].

#### 3.2.4. Optimization Tools

Response surface methodology (RSM) is a mathematical and statistical tool that allows us to determine the optimal value for a series of independent variables in order to maximize a response variable and construct a model with appropriate factors [122]. Several authors have investigated the influence of C/N ratio, TS, and biochar dose on methane yield using RSM [119,122,123]. All these works studied the anaerobic co-digestion (ACoD) of manures and lignocellulosic residues and determined similar values for both the optimal operational parameters and the maximum theoretical methane yield, which was later confirmed experimentally. However, the order of importance of the parameters and the interactions between them differed among studies. Other works have optimized biochar dose, substrate loading, inoculum loading, and substrate-to-inoculum ratio [111,115,121], and found notable interactions between biochar dose and said loadings.

A few studies have compared RSM and ANN using several statistical indicators. The best method depends on the case conditions, as there are studies reporting a better performance of RSM [119] and ANN [115], but both tools obtain very satisfactory results (R^2^ > 0.9) in terms of reliability and accuracy. Last, structural equation models (SEMs) can be used to investigate the direct and indirect effects of interactive factors. They have been used to identify the key operational parameters affecting VFA production [124] and antibiotic resistance gene transfer [61,64].

### 3.3. Integration with Other Technologies

The conversion of wet wastes to bioenergy has become a promising approach to mitigate waste management emissions and energy depletion, while promoting sustainability and a circular economy [13]. In this framework, several technology combinations have been proposed to maximize the recovery of energy and value-added products and establish a closed loop model for waste treatment.

#### 3.3.1. AD and Pyrolysis

There is considerable research regarding the integration of AD and thermal processes (e.g., pyrolysis, gasification, and hydrothermal carbonization). This section will focus on the integration of AD and pyrolysis (AD-Pyr), as it is the main process used for biochar production, but the comparison of all three coupling scenarios can be found in Pecchi & Baratieri [127]. Overall, there are three strategies for AD-Pyr integration: (1) supplementing biochar to the AD system to improve its stability and efficiency, and performing in situ/ex situ biogas purification; (2) feeding the aqueous bio-oil of pyrolysis to the digester; (3) pyrolyzing the solid digestate to produce biochar [19,127]. Biochar’s beneficial impacts on the AD process have been discussed in Trend 1 and is the strategy most reported in the literature. Aqueous pyrolytic liquid (APL) contains soluble substances that can be degraded into biogas, but also toxic compounds that inhibit AD past a concentration limit [128,129]. Some solutions to avoid this issue have been the ACoD of APL with other organic wastes [130], APL pre-treatment [131,132], and biochar supplementation [132], which merges scenarios 1 and 2. Last, biochar production through thermochemical processes is a new valorization strategy for digestate diverting from soil amendment, which can allow us to extract additional energy (gaseous and liquid combustibles) from lignocellulose and other recalcitrant components of organic waste that are not converted into biogas during AD [127,133].

**Figure 2:**
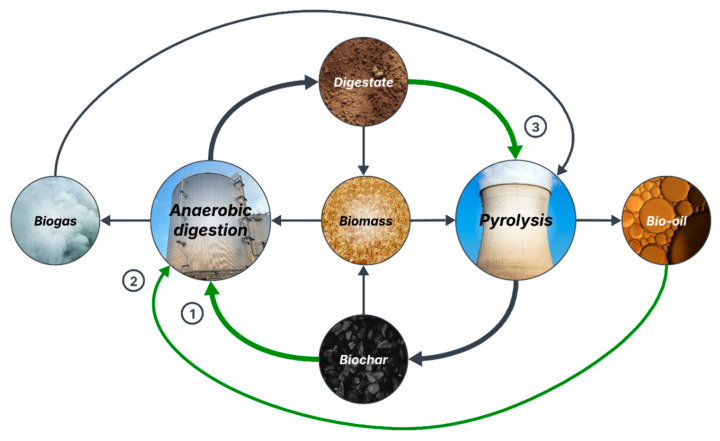
Proposed circular economy model for zero-waste management of organic waste by integrating anaerobic digestion and pyrolysis (1: biochar supplementation to AD, 2: digestion of pyrolysis bio-oil, and 3: biochar production from digestate).

Based on the studies previously discussed and following a zero-waste approach, an innovative circular economy model for organic waste management is proposed in Figure 2. This is, to the knowledge of the authors, the most complete model for AD-Pyr integration to date. In this model, biomass is treated through AD to produce biogas, which is used in pyrolysis, and digestate, which is pyrolyzed into biochar and added to AD. The residual bio-oil from pyrolysis can be recirculated to AD, while biochar and digestate can be applied separately or combined as soil amendment to grow new biomass [18,19]. The implications of biochar presence in the digestate for soil amendment applications will be discussed in Section 4.2.

#### 3.3.2. AD and Electrochemical Systems

AD can also be coupled with electrochemical systems, specifically microbial electrolytic cells (MECs). In a typical MEC, the oxidation of organic waste generates electrons in the anode; these are transported by an external power supply to the cathode and used to reduce H^+^ and CO_2_ to H_2_ and CH_4_, respectively. Combined AD-MEC systems favor the enrichment of electroactive microorganisms, promote interspecies electron transfer, accelerate substrate degradation, and enable biogas in situ purification through CO_2_ to CH_4_ conversion, all which results in an increased energy recovery [134]. Yan et al. reported a drastic increase in biogas production (76.8%), methane purity (61.11%), and solids degradation (29.9%) in an AD-MEC compared with the control [135]. The supplementation of biochar can further improve the performance of AD-MEC systems. For instance, Zhu et al. used magnetic biochar to enhance the biodegradation of landfill leachate [136], while Ning et al. added biochar to promote in situ CO_2_ bioconversion [137]. Wu et al. used Fe/Mn-modified biochar to magnify the electrochemical pretreatment of sewage sludge prior to AD, aimed at solubilizing EPS and other components [96], showing a different combination of MEC-AD-biochar. Biochar has also been used to fabricate electrodes for direct carbon fuel cells (DCFCs) and microbial fuel cells (MFCs) due to its high electrochemical conductivity, but its properties are generally insufficient to meet industrial standards [103].

## 4. Trend 3: Towards the Application of Biochar Addition to Anaerobic Digestion at Full Scale

Biochar supplementation to AD is still far from being a widely applied technology at large scale, even though some cases can be found [138]. The following studies focus on key topics that must be addressed in order to scale up this technology to full-scale organic waste treatment plants. The first step is operating the AD process with biochar supplementation under continuous conditions, which may pose some challenges. A major issue in this regard is the effects that the addition of biochar to the AD system might have on the characteristics of the digestate generated in the process, as it is often applied to the soil as fertilizer. All these factors, among others, become relevant when determining whether biochar-amended AD is both environmentally beneficial and techno-economically feasible. Ultimately, this will determine the chances of this technology being incorporated into organic waste management facilities.

### 4.1. Continuous Operating Conditions

The operation of AD with biochar addition under continuous or semi-continuous conditions is a crucial step towards the implementation of this technology in full-scale waste treatment facilities. Around 30 case studies of semi-continuous AD systems with biochar supplementation can be found in the literature, dating back to 2014. The most relevant details of these cases have been summarized in Table 4. The AD systems treat a wide variety of organic waste, including food waste, the organic fraction of municipal solid waste (OFMSW), sewage sludge, industrial wastewater, diverse manures and slurries, and lignocellulosic residues. Some works use synthetic preparations of organic wastes [39,139,140]. Mesophilic AD (35–40 °C) is more studied than thermophilic AD (over 50 °C), as it occurs in most cases. Generally, the working volume of the reactors range between 1 and 20 L, with some going up to 50 L [139,141,142]. The biggest reported case of such a system is a 1000 L reactor (700 L of working volume) treating food waste under thermophilic conditions [143]. The vast majority of the AD systems are single stage, but some are two-stage systems with separate hydrolysis and methanogenesis reactors [39,144,145,146,147].

Regarding the biochar, pyrolysis is the most common production process, and lignocellulosic biomass is the most common feedstock, although there are also biochars produced from sewage sludge [30,148], digestate [82], manure [142], and algal biomass [149]. A hot-spot of research in this field is the lack of consensus on the optimal biochar dose and whether it should be reported in absolute units (g, g d^−1^), based on working volume (g L^−1^) or based on total solids (TSs) or volatile solids (VSs) (% *w*/*w* TS, % *w*/*w* VS). Another important question that arises when AD systems are operated under semi-continuous conditions is how to ensure that biochar and its beneficial effects persist over time. Many works do not consider the loss of biochar, while others do replenish the lost biochar in order to maintain the desired dose inside the reactor [82,145,149,150]. These inconsistencies between studies on the dose and method of biochar addition complicate their accurate comparison. The efforts that were made to standardize discontinuous BMP assays [151] could now be used for semi-continuous operation conditions to solve this problem.

Finally, semi-continuous AD systems can be used to test several operational parameters like OLR [37,143,149,150], hydraulic retention time (HRT) [139,146,152], the concentration of inhibitors [37,132,144], or biochar doses [144,146,153]. The fact that these systems can be operated for longer periods of time than BMP batch tests allows us to study the medium- and long-term effects of biochar supplementation, like changes in microbial populations, metabolic pathways, enzyme activity, and functional gene expression [37,39,82,146], all which were discussed in Trend 1.

**Table 4:** Case studies of biochar supplementation in anaerobic digestion systems operating under continuous conditions (the reactor volumes in parenthesis correspond to the working volume of the reactors, fields marked with a dash are unknown).

Substrate(s)	Temperature (°C)	Reactor Volume (L)	HRT (Days)	Biochar Production	Biochar Dose	Reference
FW	55	1.1 (0.8)	-	Wood chips (800 °C)	0, 5, 10 g L^−1^	[154]
FW	55	2.3 (1.8)	15	Wood (-)	2 g L^−1^	[155]
FW	37	20 (-)	30	Digestate (565 °C)	15.52 g L^−1^	[82]
FW	10–20	H 150 (-), M 100 (-)	H (3–20), M (7–21)	Pine saw dust (650 °C)	10 g L^−1^	[147]
FW	55	1000 (700)	30	Waste wood pellets (700–800 °C)	7.5–15 g L^−1^	[143]
Synthetic FW	37	H (15), M (30)	H (1), M (3.1)	-	1, 3, 5 g L^−1^	[39]
Synthetic OFMSW	37	30 (18)	10–50	Wood waste (700 °C)	30 g L^−1^	[139]
Banana waste	35	3.5 (-)	28–42	Soft wood waste (480–540 °C)	10% (dry mass)	[156]
Corn stalk	45	16 (-)	30	Cottonwood (700 °C)	8, 16, 24 g L^−1^	[157]
Corn straw	38	16 (-)	20–40	Corn straw and coconut shell (600 °C)	4% (TS substrate)	[152]
SS	55	H 0.5 (0.4) M 0.5 (0.4)	H (5–15), M (13–30)	Corn stover and pine (-)	0.25–1 g d^−1^	[146]
Primary SS	55	1.8 (1.5)	15	Corn stover (600 °C)	1.82 g g^−1^ TS	[113]
Mixed SS	37	- (3)	20	SS (550 °C)	10 g L^−1^	[148]
Dewatered SS	35	- (5)	25–50	Bamboo (700 °C)	10 g L^−1^	[158]
Swine waste	38	- (3)	2–7	Wheat and corn straw (500 °C)	150 g	[159]
Chicken manure	35	10 (8)	20	Orchard waste (550 °C)	4.97% (TS substrate)	[150]
Dry chicken manure	35	10 (8)	20	Discarded fruitwood (550 °C)	5% (TS)	[160]
Dairy manure	39	40 (30)	23	Spruce and pine wood (550–680 °C)	10 g L^−1^	[141]
Industrial wastewater	37	2 (1.8)	30–45	Dried biosolids (600 °C)	2:1 (based on g of TS)	[161]
Aqueous pyrolysis liquid	40	1 (-)	-	Corn stalk pellets (400 °C)	24 g	[132]
OFMSW and garden waste	38	1 (0.6)	-	Wood pellets (700 °C)	0–45 g L^−1^	[153]
Synthetic FW and dewatered SS	35	- (0.15)	5–50	Sawdust (500 °C)	15 g L^−1^	[140]
Synthetic FW and SS	35	2 (-)	-	Wheat straw pellets (550 °C)	10 g L^−1^	[162]
FW and dewatered SS	37	18 (14.5)	45	Dewatered SS (300 °C)	0.075% (VS substrate	[30]
FW and algal biomass	35 and 55	1 (0.7)	-	Algal biomass (500–600 °C)	15 g L^−1^	[149]
Vegetable, garden and fruit waste, and chicken manure	37	4.5 (-)	-	Green waste and frass (450 °C)	5% *w*/*w* (substrate)	[163]
Fruit and vegetable waste, and laying hen manure	37	- (50)	30	Fruit and vegetable waste, laying hen manure, and wood pruning waste (450 and 550 °C)	1 g L^−1^	[142]
Corn stover and chicken manure	-	2.5 (2)	10–20	Rice husk (550 °C)	10 g L^−1^	[164]
Corn straw and pig manure	37	H (5), M (7.5)	H (1), M (25)	Corn straw (550 °C)	2–10% (VS substrate)	[144]
Wheat straw and swine manure	35	10 (8)	20–25	Waste apple wood (550 °C)	10 g L^−1^	[37]
Grass silage and cattle slurry	37 and 55	-	H (4), M (16), single stage (20)	Waste wood (700 °C)	10 g L^−1^	[145]

FW: food waste; H: hydrolysis reactor; HRT: hydraulic retention time; M: methanogenesis reactor; OFMSW: organic fraction of municipal solid waste; SS: sewage sludge.

### 4.2. Biochar Effects on Digestate Characteristics

Digestate is a sustainable alternative to mineral fertilizers and can act as a reliable organic soil amendment when properly handled and managed [165], being in accordance with current legislation (EU Regulation 2019/1009). Biochar has also been widely used as fertilizer [19] and, therefore, its incorporation into digestate would be expected to be advantageous. The impregnation of digestate on various biochars for the preparation of biochar-based fertilizers successfully increased N, P, and K concentrations, promoting controlled nutrient release and plant nutrition [166]. The nutrient adsorption capacity of biochar was correlated with some of its properties (atomic O/C and HC ratios, CEC, and SSA), which in turn determined its slow-release capacity [166]. The co-application of digestate and biochar has been found to enhance plant growth over digestate application alone on multiple occasions [167,168], evidencing this dual stimulation. Viaene et al. found that biochar addition during AD resulted in a digestate with a higher C and N content, and mixing biochar with the digestate reduced CO_2_ emissions once applied to the soil, while none of the biochar-amended digestates triggered N release as NH_3_ or N_2_O [163].

Some semi-continuous AD assays have studied the characteristics of digestate after long-term biochar supplementation. These works found that biochar released important elements (N, P, K, Ca, Mg, Fe) that increased the nutrient content of the digestate [146,149,162]. Tariq et al. observed that biochar treatments producing higher biogas yields also had digestates with higher nutrient contents and hypothesized that the promotion of microbial activity enhanced the mineralization of organic matter and, subsequently, the amount of nutrients available [169]. Biochar could also help immobilize heavy metals [170], thus reducing their bioavailability and associated toxicity, although more research is needed on this matter, especially on its implications for metal-modified biochars. Investigation on this matter is of high importance to ensure that the final digestate can be used as fertilizer safely and in compliance with the legislation in force.

### 4.3. Technical and Environmental Assessment

Several scenarios have been investigated and compared from an environmental and techno-economical point of view. This includes the comparison of conventional waste management practices (e.g., landfilling and direct soil application) with biological treatments like AD, and the integration of AD with other technologies, with the combination of AD with thermochemical processes for biochar production being the most common approach (Section 4.3.1). The feasibility of biochar use in AD at both levels is indispensable for its upscaling and eventual application in real-scale waste treatment facilities.

#### 4.3.1. Life Cycle Assessment

Life cycle assessment (LCA) is a tool to assess the inputs, outputs, and potential environmental impacts of a product system throughout its life cycle [171]. It considers several impact groups (e.g., eutrophication, acidification, ozone depletion, human toxicity, ecotoxicity, etc.) and calculates the overall global warming potential (GWP) in terms of CO_2_ equivalents (CO_2eq_) emitted per functional unit. Uddin et al. compared the environmental impact of beef manure digestion with and without biochar supplementation and found that biochar addition reduced GWP by 32% from −0.033 to −0.043 kg CO_2eq_ per MJ of biomethane produced. Both scenarios presented net negative emissions, proving the sustainability of AD, which were enhanced with biochar due to N_2_O emissions avoided from the soil application of biochar-amended digestate compared with regular digestate [172]. Tian et al. reported a reduction of 42 kg CO_2eq_ per t of food waste treated and an improvement in all nine impact categories with biochar addition [173]. On the contrary, Yang et al. reported that biochar supplementation increased GWP due to the energy consumption for its manufacture, although it reduced several impact groups [156]. Moreover, when biochar transportation was considered, GWP was further increased, meaning that the environmental burden of transportation overshadowed the benefits associated with its supplementation. This highlights the importance of proximity when integrating technologies in a biorefinery approach. Mediboyina et al. evaluated the relevance of biochar dose and the final destination of the biogas generated (conversion to bioelectricity or upgrading to biomethane). Biochar addition improved the environmental sustainability of the process in all cases, generally proportional to the dose, while the biogas fate had diverse effects across impact categories [174]. The addition of biochar proved to be the only environmentally beneficial strategy to improve sludge AD on a large scale compared with different sludge pre-treatment technologies, although it was not economically viable [175].

Several studies have demonstrated that coupling AD with biochar production through thermochemical processes decreases environmental impact by avoiding the landfilling or incineration of solid digestate and increasing energy generation [176]. For instance, Mehta et al. conducted a case study in Northern Ireland and identified AD–pyrolysis (−563 kg CO_2eq_ per person) as the best treatment scenario for livestock manure and grass silage over AD (−464 kg CO_2eq_ per person) and direct land application (344 kg CO_2eq_ per person) [177]. Similarly, Hosseinian et al. established AD–pyrolysis as the best practice to manage sewage sludge in Sweden from an environmental point of view, yielding a lower GWP (−471 kg CO_2eq_) than AD and pyrolysis alone (−224 and −293 kg CO_2eq_, respectively) [178]. In conclusion, the environmental benefits of biochar and AD coupling rely on the conventional and more-polluting processes that they avoid, like the production of mineral fertilizers for soil amendment that can be substituted by digestate and/or biochar, or the generation of heat and electricity from non-renewable sources that is since covered by biofuel production from AD (biogas) and pyrolysis (syngas).

#### 4.3.2. Techno-Economic Analysis

Although biochar integration in AD has generally been proven to be environmentally beneficial, it is not always economically profitable. A techno-economic analysis (TEA) of the costs associated with the addition of biochar to AD is key to determining its applicability at large scale, but studies including a TEA are very scarce in the literature. The economic viability of the process generally depends on whether the increase in methane yield resulting from biochar addition can offset the cost of biochar itself. The price of biochar depends on the feedstock, process temperature, and scale, but is generally more affordable (0.2–0.5 USD kg^−1^) than similar additives like granular activated carbon (0.6–20 USD kg^−1^) [10]. The balance between the dose of biochar added and the resulting improvement in methane yield usually determines whether biochar addition is economically feasible [172] or not [179].

There are several ways to increase the economic benefits of the process. Depending on the regulations in force at each place, solid digestate can be sold as soil conditioner for a small sum (0.035 USD kg^−1^) [180], which could be higher for biochar-amended digestate due to biochar’s beneficial effects on crops [181]. Other sources of income are the carbon credits given for the use of biogas instead of fossil fuels or the removal of dangerous pollutants. In a recent study conducted by Hasanan et al., biochar enhancement of methane yield and 1,2-dichloroethane removal increased the profits from selling biogas, pollution reduction, and carbon credits, which made the scenario economically feasible (6.24 years payback period, with 10 years project lifetime), when otherwise it was not [40]. The same occurred in the case study of Hafez et al., where biochar addition was the only economically feasible scenario for the treatment of paper mill sludge laden with 2-chlorotoluene [182]. Another strategy that is gaining interest in maximizing the economic profitability of the process is to valorize the digestate for biochar production, which has a higher market price. This idea has been tested in various scenarios including combinations of ACoD–pyrolysis [183,184], adsorption–ACoD–pyrolysis [185], phytoremediation–ACoD–pyrolysis [186], and hydrodynamic cavitation–AD–pyrolysis [187]. Apart from being sold, biochar could be reused in situ for AD performance improvement, which would close the material loop in organic waste treatment. To the knowledge of the authors there are no TEAs assessing the production of biochar from digestate and its application in AD simultaneously.

## 5. Current Limitations and Future Prospects

This review investigated the most significant lines of research regarding the use of biochar to improve AD. From this discussion, relevant knowledge gaps and challenges can be defined (Table 5), which should consequently be addressed in future studies. First, the knowledge generated on the mechanisms behind biochar-enhanced AD should be exploited to identify the circumstances in which biochar addition may be most effective in improving AD performance. Future works could further investigate the role of biochar’s physicochemical properties on these mechanisms to reveal, for instance, how biochars with different porosities or functional groups may enrich different microbial communities, upregulate different functional genes, or promote different metabolic pathways. Following the same reasoning, biochar modifications should aim to improve the most advantageous properties for each case. By using all the available information, the most appropriate biochar type could be selected for each specific scenario, considering both the characteristics of the material and the idiosyncrasies of the AD system. Consequently, AD performance could be improved by tackling more specific goals than simply increasing methane yield (e.g., avoiding VFA accumulation with highly biodegradable wastes, accelerating degradation with poorly biodegradable wastes, promoting pollutant elimination in inhibited systems, suppressing ARGs, etc.). For this purpose, multi-omics methods revealing associated mechanisms would be of great interest. Another valuable tool is modeling and optimization, which could be further improved by creating more complete models and applying them to various scenarios (e.g., different feedstocks, functionalized biochars, the presence of inhibitory conditions, etc.). Some understudied scenarios that could benefit from this are psychrophilic AD (PAD) and anaerobic membrane bioreactors (AnMBRs). A major milestone in the modeling field would be the application of the ADM1 model to biochar-enhanced AD. Again, the information generated from these models should be used to guide decision-making in order to select the best biochar type for each AD system.

A topic that needs to be investigated in more depth is the consequences of biochar modifications on both the AD process and the characteristics of the digestate. This notion should be included in all future work using functionalized biochars, especially those containing metals, to avoid any undesired effects such as the inhibition of microbial activity or the release of metals into the soil if the digestate is to be applied as fertilizer. Another area requiring further work is the application of biochar to AD systems operating under semi-continuous conditions. In this sense, a consensus should be reached on the units in which biochar dosage is reported in order to facilitate comparison between studies. The persistence of biochar and its effects in the AD system over time, as well as its distribution inside the reactor and potential reuse, are an almost unexplored aspect of research on the subject. Other questions that should be covered in the future are how to overcome the loss of biochar during long-term operations, and how the decrease in biochar particle size due to agitation and mixing can affect system performance. Finally, LCA and TEA have shown that the supplementation of biochar to AD might only be both environmentally beneficial and techno-economically viable under certain circumstances. The applicability of biochar addition to AD on a large scale is subject to many factors (e.g., biochar price and transportation, increase in methane yield and purity, the degradation of pollutants, the reuse of energy and heat streams, the replacement of mineral fertilizers by digestate, etc.). Some are not directly related to science but should still be considered from the early stages of research to avoid spending time, resources, and effort on unfruitful research. The integration of AD and pyrolysis appears as a promising strategy for sustainable organic waste management that would fulfill the modern purposes of circular economy and zero-waste models. In conclusion, future research should focus on further exploring the aforementioned trends and integrating the knowledge generated from each of them. Solving current challenges, such as understanding the consequences of biochar modifications in AD and digestate and reaching a consensus on biochar dosage, together with approaching biochar applicability in AD from a global perspective, is of utmost importance for research in this field to progress and prosper.

## 6. Conclusions

In recent years, research concerning the use of biochar to improve AD has increased exponentially and its expansion has given rise to numerous and diverse research lines. Findings at genetic, enzymatic, metabolic, and microbial levels have helped to elucidate the mechanisms behind the beneficial effects of biochar on AD (e.g., increase in methane yield and purity, the alleviation of VFA and ammonia inhibition, pollutant removal, etc.), which are strongly correlated with each other. The positive impact of biochar on AD can be further maximized by modifying its properties, using modeling and optimization tools, and integrating AD with other technologies. However, further research is needed in the case of functionalized biochar to fully comprehend the consequences of these modifications on the AD system and the characteristics of the digestate. Regarding the application of biochar to AD systems under continuous operation, a consensus is needed on the optimal dose and addition method, as well as a deeper understanding of biochar behavior during long-term operations, which are crucial challenges to be addressed in the future. Although biochar supplementation generally improves the environmental sustainability of AD, its application at large scale might only be techno-economically feasible in certain scenarios, like the treatment of specific polluted streams and/or the combination of AD and pyrolysis in a closed-loop organic waste treatment model. The trends and challenges discussed in this review define the way forward for future research in this field. This is of great relevance given the current rise in AD and could be decisive for this technology to become a pillar of renewable energy in Europe and worldwide.

## 7. Materials and Methods

### 7.1. Literature Search

The references cited in this review were gathered from Scopus^®^, as it is one of the largest and most relevant academic databases, especially in the scientific field. An initial search for reviews was made in order to identify the latest trends in research related to biochar use in AD. The search query used was as follows: TITLE-ABS-KEY (biochar AND anaerobic digestion) AND TITLE (new OR recent OR emerging OR future OR trends OR perspectives OR challenges OR advances). Once the main topics of the review were selected, specific searches were made for each one of them and their respective subtopics. The search queries used in this case were as follows: TITLE-ABS-KEY (biochar AND anaerobic digestion AND “keyword”), where the “keyword” was selected for each topic of interest (interspecies electron transfer, quorum sensing, antibiotic resistance genes, biochar engineering, model, semi-continuous operation, digestate, techno-economic analysis, and life cycle assessment).

### 7.2. Selection Criteria

The first criterion was to limit the publication year to the last 5 years (2020–2024), as the main goal of this review was to gather the latest and most novel lines of research on the topic. For the initial prospection on relevant trends, only review articles were selected, whereas for the subsequent searches on the selected topics, both reviews and research papers were included. Lastly, papers whose focus diverted greatly from the main topic, which is biochar use as an additive to improve AD, were discarded. Some papers that did not fit the aforementioned criteria but were considered of interest were also selected. The articles were downloaded using the “CVS” format, processed using Microsoft Excel^®^, and reviewed in order to identify and explore the main trends in the current research on biochar use in AD.

## Figures and Tables

**Table 2:** Activation and modification methods used to generate functionalized biochar types for AD applications.

Type	Method	Reported Improvements in Biochar Characteristics	References
Physical	Steam, CO_2_, ozone, MWAP	Higher SSA and porosity Introduction of oxygen-containing functional groups	[71,72,73,74]
Chemical	H_2_O_2_	Higher SSA and porosity Introduction of oxygen-containing functional groups	[75]
	Acid agents (H_3_PO_4_, H_2_SO_4_, HNO_3_, KH_2_PO_4_)	Higher SSA and porosity Introduction of acid and oxygen-containing functional groups	[64,76,77,78,79,80,81]
	Alkaline agents (KOH, NaOH, K_2_CO_3_)	Higher SSA and porosity Introduction of oxygen-containing functional groups Greater aromatization of biochar structure	[82,83,84,85]
	Metallic agents (Fe, Co, Mn)	Improved redox properties In the case of nZVI, CO_2_ to CH_4_ conversion In the case of magnetic biochar, simple recycling	[25,29,45,76,86,87,88,89,90,91,92,93]
	Heteroatom doping (N, S)	Introduction of N- and S-containing functional groups Improved EC and electron transfer	[77,94]
	Combinations	Synergistics effects over single modifications	[95,96,97,98,99]
Biological	Microorganisms	Immobilization of microbial communities Protection against inhibitory conditions	[100,101,102]

MWAP: microwave-assisted pyrolysis.

**Table 3:** Modeling and optimization tools used to assist biochar applications in anaerobic digestion (fields marked with a dash have no reported cases).

Tool/Technology		References
Mathematical models	Mechanistically inspired models Anaerobic Digestion Model No. 1 (ADM1)	-
	Kinetic models First order model (FOM) Logistic function model (LFM) Modified Gompertz model (MGM) Transference function model (TFM)	[61,73,108,109,110,111,112,113]
	Phenomenological models (machine learning) Artificial neural networks (ANN) Random forest (RF) Automated machine learning	[114,115,116,117,118,119,120]
Optimization tools	Response surface methodology (RSM)	[111,115,119,121,122,123]
	Structural equation model (SEM)	[61,64,124]

**Table 5:** Critical research gaps and priority topics for future investigations in biochar use to improve anaerobic digestion.

Identified Trends	Topics to Be Addressed in Future Works
Mechanisms	Further investigate the importance and role of specific biochar properties for each mechanism. Apply multi-omics approaches to elucidate associated mechanisms. Assess biochar effects in understudied scenarios like PAD and AnMBR.
Novel tools	Tailor biochar properties to the requirements of each system. Extend mathematical models to diverse scenarios, including the application of ADM1 to biochar-enhanced AD. Explore integration scenarios.
Applicability	Define the impacts of biochar modifications on AD performance and digestate characteristics. Find a consensus on the dosing units for biochar-supplemented reactors under continuous operation. Address biochar distribution, persistence, and potential reuse. Incorporate technical and environmental feasibility perspective in more studies.

## Data Availability

This article does not include new data.

## Abbreviations

The following abbreviations are used in this manuscript:

AAN	Artificial Neural Network
ACK	Acetate Kinase
ACoD	Anaerobic Co-Digestion
AD	Anaerobic Digestion
ADH	Ethanol Dehydrogenase
ADM1	Anaerobic Digestion Model No. 1
AHL	Homoserine Lactone
AI-2	Autoinducer-2
AIP	Antipeptide
AK	Adenylate Kinase
ANFIS	Adaptive Neuro-Fuzzy Interference System
AnMBR	Anaerobic Membrane Bioreactor
APL	Aqueous Pyrolytic Liquid
ARGs	Antibiotic Resistance Genes
ATP	Adenosine Triphosphate
BK	Butyrate Kinase
BMP	Biochemical Methane Potential
CAs	Carbon-Based Additives
CAZymes	Carbohydrate-Active Enzymes
CEC	Cation Exchange Capacity
CO_2eq_	CO_2_ Equivalents
Co-di-GMP	Cyclic Diguanosine Monophosphate
CoF_420_	Coenzyme F_420_
CoM	Coenzyme M
DCFCs	Direct Carbon Fuel Cells
DIET	Direct Interspecies Electron Transfer
DoE	Design Of Experiments
DSF	Diffusible Signal Factor
EC	Electrical Conductivity
EPS	Extracellular Polymeric Substances
ETS	Electron Transport System
FOM	First-Order Model
GWP	Global Warming Potential
HOM	Homoacetogenesis
HRT	Hydraulic Retention Time
IIET	Indirect Interspecies Electron Transfer
IWA	International Water Association
KEGG	Kyoto Encyclopedia of Genes And Genomes
LB-EPS	Loosely Bound Extracellular Polymeric Substances
LCA	Life Cycle Assessment
LFM	Logistic Function Model
MECs	Microbial Electrolytic Cells
MFCs	Microbial Fuel Cells
MGE	Mobile Genetic Element
MGM	Modified Gompertz Model
MWAP	Microwave-Assisted Pyrolysis
NprX	Signaling Peptide
nZVI	Nano Zero-Valent Iron
OFMSW	Organic Fraction of Municipal Solid Waste
OLR	Organic Loading Rate
PAD	Psychrophilic Anaerobic Digestion
POR	Pyruvate Ferredoxin Oxidoreductase
PTA	Phosphate Acetyltransferase
PTB	Phosphate Butyryltransferase
QS	Quorum Sensing
RF	Random Forest
RSM	Response Surface Methodology
SBO	Syntrophic Butyrate Oxidation
SPO	Syntrophic Propionate Oxidation
SSA	Specific Surface Area
SVM	Support Vector Machine
TB-EPS	Tightly Bound Extracellular Polymeric Substances
TCA	Tricarboxylic Acid (Cycle)
TEA	Techno-Economic Analysis
TFM	Transference Function Model
TSs	Total Solids
VFAs	Volatile Fatty Acids
VSs	Volatile Solids
